# Cold beverage-induced vasovagal syncope in a healthy young adult man: a case report

**DOI:** 10.1186/s13256-020-2358-3

**Published:** 2020-02-28

**Authors:** Ognjen Arandjelović

**Affiliations:** University of St Andrews, North Haugh, St Andrews, Fife, KY16 9SX UK

**Keywords:** Fainting, Swallowing, Loss of consciousness, Vagus nerve, Parasympathetic

## Abstract

**Background:**

Swallowing-induced syncope is rare and there are few case reports of it in the existing medical literature. Even rarer are instances involving young and healthy individuals, with no existing pre-conditions or apparent risk factors. Hence the value of such case reports in understanding the phenomenon better and potentially inferring patterns of practical interest is significant; here we describe an unusual case of a swallowing-induced syncope in a young, healthy, and active white man.

**Case presentation:**

A healthy 32-year-old white man experienced a syncopal episode following the ingestion of a cold carbonated beverage on a hot day. He rapidly recovered consciousness and save for mild lightheadedness all ill effects disappeared within minutes. On examination no concerns were detected and he was discharged, with the cause being ascribed to esophageal stimulation effected vagus nerve overactivation.

**Conclusions:**

The suddenness and unpredictability of swallowing-induced syncope make it a potentially dangerous condition, with risks both to the patient as well as, depending on the context, others. However, it is poorly understood due to its infrequency. The present case report adds to the body of much needed evidence which should help facilitate an improved understanding of the phenomenon.

## Introduction

Vasovagal syncope induced by swallowing is rare. This makes it difficult to study and collect reliable evidence on causes, populations at risk, symptoms, recovery, and so on. Considering this practical challenge and the high potential of serious consequences that a sudden loss of consciousness can have, it is imperative to encourage diligent reporting of case studies as a means of systematically collecting information on the phenomenon so that it is better understood by practitioners and individuals at risk. The present article contributes the following: (i) it adds to the body of evidence through a description of a particularly unusual case of swallowing-induced syncope, and (ii) it highlights the need for reports of this nature both for the specific condition at hand as well as in general for rare conditions which share the same practical challenge of data scarcity.

## Case presentation

A healthy 32-year-old white man was examined after suffering from a syncope episode. The incident took place following a visit of 2–3 hours’ duration to a wildlife park on a sunny and hot day (approximately 37 °C in the shade) in a café while he was resting and consuming a cold carbonated beverage. He reported that he had been feeling fine until he swallowed the first mouthful of the beverage when he experienced a sharp painful sensation in his chest, followed by a blurring of vision. Within a few seconds he lost consciousness. Due to the quick reaction of one of the individuals accompanying him, he (seated at the time) was prevented from hitting his head and was slowly lowered to the ground. He quickly regained consciousness and had no recollection of fainting. Except for a feeling of lightheadedness, all ill effects disappeared promptly.

At the time of examination, approximately 30 minutes following the syncope, his body mass was 102 kg and height 188 cm with a body mass index (BMI) of 28.9. He self-reported an active lifestyle and regular involvement in challenging resistance training (4–5 times a week), corroborated by his high lean body mass (LBM) and low body fat (BF) (8%); there was no history of recreational or performance-enhancing drug use, and no use of medications (either prescription or over-the-counter). He had experienced no prior episodes of syncope, although he did note experiencing sensations similar to those leading to the present episode upon swallowing cold carbonated beverages in the past; the frequency of these sensations was not deemed unusual or as warranting further investigation (self-estimated as being less frequent than once per annum). At the time of examination, his blood pressure was 128/77 mmHg and his resting heart rate (RHR) was 74 beats per minute (bpm). He reported a history of low blood pressure in his family, both on the maternal and paternal sides, and his somewhat elevated systolic measurement was attributed to the weather conditions and the overall arousal concerning the episode.

Taking into account the entirety of the context and presented symptoms, that is, our patient’s state at the time of examination, health condition, and symptoms, and the one-off nature of the incident, the syncope was attributed to vagus nerve overactivation effected by esophageal stimulation. In a 5-year follow-up, he reported no recurrence of syncope (swallowing induced or otherwise) but confirmed several more instances of the aforementioned unpleasant sensations (including sharp transient pain, blurry vision, and lightheadedness but no actual loss of consciousness) all associated with the swallowing during the ingestion of cold carbonated drinks. Computed tomography (CT) scans of his brain and thorax revealed no abnormalities, and tests of his thyroid revealed normal functioning.

## Discussion and conclusions

The reports of swallowing-induced syncope in the existing medical literature are scarce and the general consensus is that such episodes are in fact rare [1]. What is more, the swallowing syncope is usually observed in individuals with underlying abnormalities [2] who usually attract attention due to their experiencing repeated Adams–Stokes attacks [3, 4]. Most of these reports also involve older individuals, aged over 60 years. These patterns are in sharp contrast with the present case which involves a young, healthy, and active individual, with no obvious risk factors or readily apparent predisposing conditions. Few such examples have been reported in the existing peer-reviewed medical literature [5, 6], highlighting the value of documenting the present case.

## Data Availability

Not applicable.
